# Enzyme histochemistry of cecal lymphoid tissue during prenatal period of buffalo

**DOI:** 10.14202/vetworld.2016.1006-1011

**Published:** 2016-09-24

**Authors:** Kritima Kapoor, Opinder Singh

**Affiliations:** Department of Veterinary Anatomy, Guru Angad Dev Veterinary and Animal Sciences University, Ludhiana, Punjab, India

**Keywords:** buffalo, cecum, histoenzyme, lymphoid tissue, prenatal

## Abstract

**Aim:**

This study was designed to elucidate the histoenzymic distribution of enzymes, i.e., phosphatases, oxidoreductases, dehydrogenases, and diaphorases in cecal lymphoid tissue during its development in the prenatal period.

**Materials and Methods:**

The study was conducted on cecum of 15 buffalo fetuses ranging from 16 cm curved crown-rump length (CVRL) (100 days) to 100 cm CVRL (full term). The fetuses were categorized into three groups based on their CVRL.

**Results:**

In Group I, the distribution of enzymes was uniformly weak in developing villi-like projections in cecum and completely absent from submucosa. In Group II, the enzymes showed a moderate to strong activity in epithelium lining tunica mucosa which progressively decreased as the fetus progresses toward late gestational age. However, the intense activity of enzymes was observed in developing lymphoid tissue in this group. In Group III, distribution of enzymes reduced in tunica mucosa of cecum with advancing age, whereas the intense activity was noticed in the developed lymphoid tissue complex.

**Conclusion:**

The distribution of enzymes was completely absent from submucosal region in cecum of Group I as there was no lymphoid tissue development at this age. In Group II, the enzymes showed a moderate to strong activity in epithelium lining tunica mucosa which progressively decreased toward late gestational age but an intense activity was observed in developing lymphoid tissue. In Group III, distribution of enzymes reduced in tunica mucosa with advancing age with intense activity noticed in the developed lymphoid tissue complex.

## Introduction

The alimentary tract is comprised of the highest microflora; as a result, it is important to differentiate potentially harmful pathogens from inhabitant normal gut microflora. Therefore, in the intestine, the gut-associated lymphoid tissue (GALT) is present that acts to protect the animal and generate an immune response against the foreign antigens entering the alimentary tract [1]. GALT is composed of lymphoid patches both in the small and large intestine. Cecum contains a part of the lymphoid tissue of large intestine. The cecal lymphoid tissue is present as solitary nodules as well as diffused lymphoid tissue (DLT). The cecal lymphoid tissue is in direct contact with the lumen, and the dome epithelium provided both a protective barrier and a route for antigen uptake from the gut [2]. The plasma cells then secrete immunoglobulin-A which is added directly into the lumen to provide an immunological response.

The presence of many enzymes including phosphatases, dehydrogenases, and esterases in cecal lymphoid tissue participate in a major role of the protection as well as in other physiological roles in the gut. Therefore, the tissue localization of these enzymes is achieved by enzyme histochemistry of these intestinal tissues. Alkaline phosphatase (AKPase) is associated with the ionic exchange across the membrane as well as for endocytosis and pinocytosis in the cells [3]. Diaphorase activity represents mitochondrial activity along with the cytoplasmic electron transport. Succinic dehydrogenase (SDH) is an essential part of Kreb’s cycle and also found in all aerobic cells. Moreover, glucose-6-phosphatase (G-6-pase) is a key enzyme which regulates the glucose mechanism in the intestine. G-6-pase is a hydrophobic protein which was observed to be embedded within endoplasmic reticulum of the luminal membrane [4].

Adequate reports have been made on lymphoid tissue of cecum of rabbit [2,5], chicken [6,7], rats [8], and goats [9]. However, very few findings are available on cecal lymphoid tissue of buffalo during prenatal life. Moreover, there is no literature available on histoenzymology of cecal lymphoid tissue in buffalo during its prenatal period. Therefore, this study was undertaken to explicate the tissue localization of various enzymes in cecal lymphoid tissue of buffalo during its prenatal life.

## Materials and Methods

### Ethical approval

This study was conducted after approval by the Research Committee and Institutional Animal Ethics Committee.

### Animals

This study was conducted on cecum of 15 buffalo fetuses belonging to different gestation periods collected from buffaloes slaughtered at different abattoirs. Immediately after collection, curved crown-rump length (CVRL) of fetus was measured in centimeters. The approximate age of fetuses was calculated using the following formula [10]:

Y=28.66+4.496X (CVRL <20 cm)

Y=73.544+2.256X (CVRL ≥20 cm)

Where, Y is age in days and X is CVRL in cm.

Depending on CVRL fetuses were divided into three groups, *viz*., Group I (CVRL between 0 and 20 cm), Group II (CVRL between 20 and 40 cm), and Group III (CVRL >40 cm) with a minimum of five samples in each group.

### Experimental design

The fresh cecum from fetuses of different age groups was immediately collected after sacrifice and stored in liquid nitrogen. The cecal tissue was subjected to cryostat sectioning at −20°C with cryostat microtome. The sections of 10-12 μm thickness were obtained on clean glass slides and were incubated for demonstration of various enzymes as described in Table-1, *viz*., phosphatases: AKPase and G-6-pase, non-specific esterase (NSE) and oxidoreductases: Oxidases: Monoamine oxidase (MAO); dehydrogenases: SDH, lactic dehydrogenase (LDH), G-6-Phosphate dehydrogenase (G-6-PD), nicotinamide adenine dinucleotide diaphorase (NADH-d) and nicotinamide adenine dinucleotide phosphate diaphorase (NADPH-d) [11].

**Table-1:** Histoenzymic methods used on cryostat sections of ileum of buffalo fetuses.

Enzyme	Substrate	Method	Reference	Incubation Time
Phosphatases				
AKPase	Naphthol AS-MX phosphate disodium salt in combination with Fast Blue RR	Simultaneous coupling azo dye method using substituted naphthols	Barka and Anderson (1963)	30 min
G-6-pase	G-6-pase and lead nitrate	Lead nitrate method	Barka and Anderson (1963)	20 min
Oxidoreductases				
SDH	Di-Na succinate	Standard method of bound enzyme by nitro BT method	Pearse (1972)	30 min
LDH	Na-DL lactate	Standard method of bound enzyme by nitro BT method	Pearse (1972)	30 min
G-6-PD	Di-Na G-6-pase	Standard method of bound enzyme by nitro BT method	Pearse (1972)	30 min
MAO	Tryptamine hydrochloride	Standard method of bound enzyme by nitro BT method	Pearse (1972)	60 min
NADPH-d	Co-enzyme (NADPH)	Standard method of bound enzyme by nitro BT method	Pearse (1972)	30 min
NADH-d	Co-enzyme (NADH)	Standard method of bound enzyme by nitro BT method	Pearse (1972)	30 min
Esterases				
NSE	Alpha-naphthol acetate	Naphthol acetate method	Barka and Anderson (1963)	10 min

AKPase: Alkaline phosphatase, G-6-pase: Glucose-6-phosphatase, SDH: Succinic dehydrogenase, LDH: Lactic dehydrogenase, G-6-PD: Glucose-6-phosphate dehydrogenase, MAO: Monoamine oxidase, NADPH-d: Nicotinamide adenine dinucleotide phosphate diaphorase, NADH-d: Nicotinamide adenine dinucleotide diaphorase, NSE: Non-specific esterase

## Results and Discussion

### Group I

In Group I, at 16 cm CVRL (100 days) weak AKPase activity was observed in epithelium of villi-like projections in the developing cecum. However, negligible activity was observed in submucosa of cecum as there was no lymphoid tissue development at this age. Similar observations were made in goats during their prenatal life [9]. The G-6-PD activity was also only observed in developing epithelium of cecum and similarly absent in submucosal region at this age. Moreover, weak SDH activity was observed in the epithelial cells of developing villi-like projections of cecum of 19 cm CVRL (114 days) fetus that was found to degenerate later. Conversely, the activity of LDH was completely absent from both epithelial cells a well as submucosa. Very feeble activity of G-6-PD, NADH-d–, and NADPH-d was observed in lamina propria of cecum at 19 cm CVRL (114 days). In addition, the tunica muscularis was also having weak activity of G-6-PD. However, insignificant activity of this enzyme was observed in submucosal area. NSE activity was not observed in cecum at this age group.

### Group II

**Figure 1:**
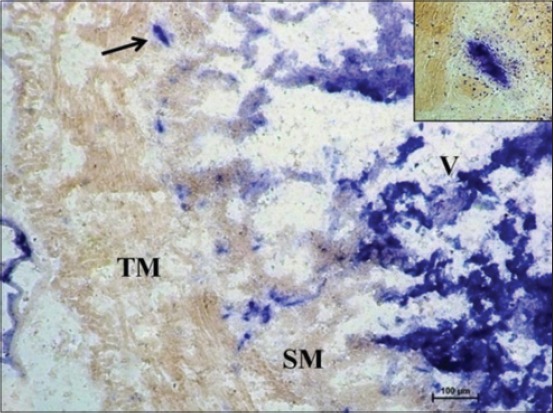
Photomicrograph of cecum of 35 cm curved crown-rump length (152 days) fetus showing strong alkaline phosphatase activity in villi-like projections (V), lymphoid aggregates (arrow, inset) in submucosa (SM) and weak activity in tunica muscularis (TM). Azodye method ×400.

**Figure 2:**
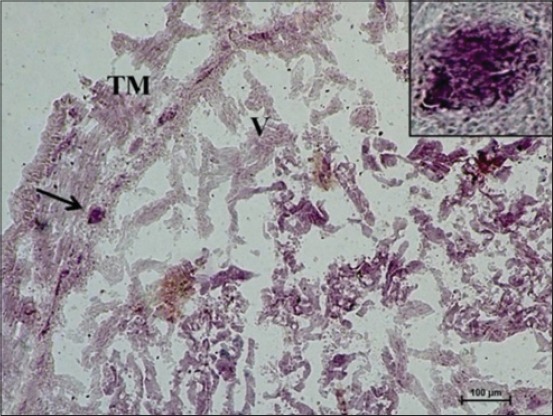
Photomicrograph of cecum of 37 cm curved crown-rump length (157 days) fetus showing weak G-6-phosphate dehydrogenase activity in apical portion of villi-like projections and strong activity in lymphocytic aggregates (arrow) in submucosal region. Nitro BT method ×100.

**Figure 3:**
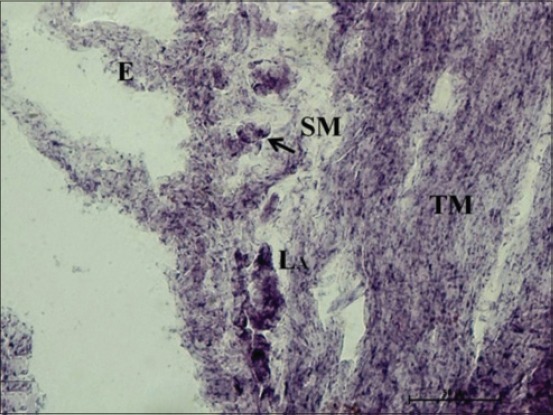
Photomicrograph of cecum of 37 cm curved crown-rump length (157 days) fetus showing moderate nicotinamide adenine dinucleotide-diaphorase activity in epithelium (E), tunica muscularis (TM) and strong activity in submucosal lymphocyte aggregates (LA) specifically in their peripheral areas (arrow). Nitro BT method ×400.

At 35 cm CVRL (152 days), a very strong AKPase activity was observed in the tunica mucosa, specifically in the epithelium of villi-like projections of cecum. At this age, the group of developing lymphocytic aggregates on antimesenteric side in submucosa also depicted strong activity for AKPase (Figure-1). However, the AKPase activity was very weak in the ileocecal region at the apical portion of the villi-like projections in this age group. Conversely, a moderate activity of this enzyme was observed in the crypts of ileocecal region. Moreover, the activity of G-6-PD was weak to moderate in apical portion of lamina propria of cecum at this age. However, the lymphocytic aggregates in submucosal region observed at this age showed moderate activity of this enzyme. The activity was however observed to be concentrated explicitly around these aggregates and being completely absent from the surrounding submucosal region. However, the activity of this enzyme in epithelium and tunica muscularis of ileocecal orifice was very weak, whereas the submucosal lymphoid aggregates showed weak to moderate G-6-PD activity. At 37 cm CVRL (157 days) also, intense AKPase activity was observed in the enterocytes lining the villous region of tunica mucosa. Moreover, the intense AKPase activity was observed specifically outlining the columnar epithelial cells lining and in the crypt region as well. An intense activity was seen in lymphocytic aggregations observed in submucosa. A weak to moderate SDH activity was observed in the villous epithelium of cecum and ileocecal orifice region. But intense activity was observed in lymphocyte accumulations of both the regions. A similar ileocecal patch was observed in the domestic animals [12]. An overall weak activity of LDH and MAO was observed in lamina propria, tunica muscularis as well as the developing lymphoid region. At this age moderate to strong G-6-PD activity was noticed in lymphocytic aggregates whereas weak to moderate activity was found in epithelium of villous-like projections and tunica muscularis of cecum (Figure-2). However, moderate to strong fine granular activity of NADH-d and NADPH-d was observed in villi. However, an intense activity of this enzyme was observed in submucosal aggregates of lymphocytes specifically in their peripheral areas (Figure-3). However, the activity of NSE enzyme was observed to be very weak in villous epithelium particularly weak activity was observed in the cytoplasm of enterocytes. However, its moderate activity was noticed in the lymphoid aggregates in submucosa.

### Group III

**Figure 4:**
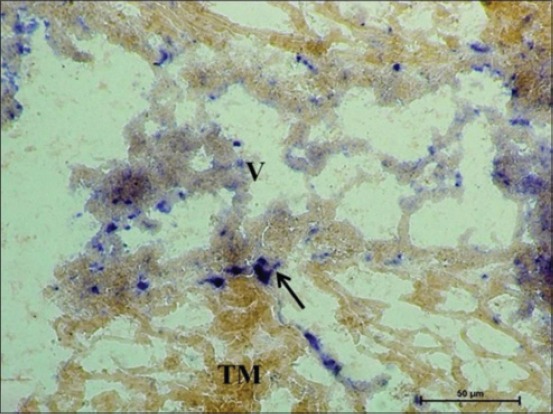
Cryostat section of 42 cm curved crown-rump length (152 days) cecum of fetus showing weak alkaline phosphatase activity in enterocytes in the villous-like projections and intense activity in submucosal lymphoid aggregates. Azodye method ×100.

**Figure 5:**
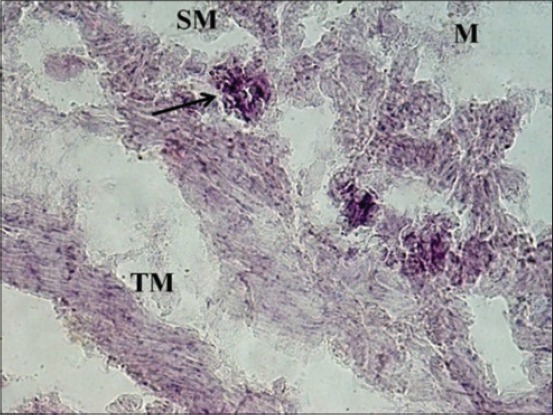
Photomicrograph of 42 cm curved crown-rump length (152 days) of fetal cecum showing weak succinic dehydrogenase activity in tunica mucosa (M), tunica muscularis (TM) and strong activity at periphery of the lymphoid primordia. Nitro BT method ×400.

At 42 cm CVRL (168 days), AKPase activity was weak in the brush border of enterocytes in the villous-like projections facing the lumen as the AKPase activity was observed to be reduced at this age group as compared to early and mid-gestational age (Figure-4). Similar observation was made in *Diplodus vulgaris* fish which stated that AKPase activity gradually decreased in a posterior direction of intestine, i.e. weaker in pyloric ceca than the anterior, posterior and middle segment of the intestine [13]. However, a moderate activity of this enzyme was seen at few places especially in the blood vessels in tunica mucosa of cecum. This enzyme might serve as a chemotactic agent, attracting blood-borne lymphocytes [14]. However, the submucosal lymphoid aggregates had intense AKPase activity (Figure-4). SDH activity was also observed to be reduced and appeared as weak activity in tunica mucosa and tunica muscularis. However, lymphoid tissue primordia developed at this age in submucosa had strong peripheral activity of this enzyme (Figure-5). Uniformly moderate LDH activity was observed in villous epithelium and on the periphery of the cells of lymphoid tissue primordia. LDH is a NAD-dependent enzyme found in cells involved in glycolytic pathway. The presence of LDH activity suggests its role in differentiation of lymphocytes in fetal life as in chicken [15].

**Figure 6:**
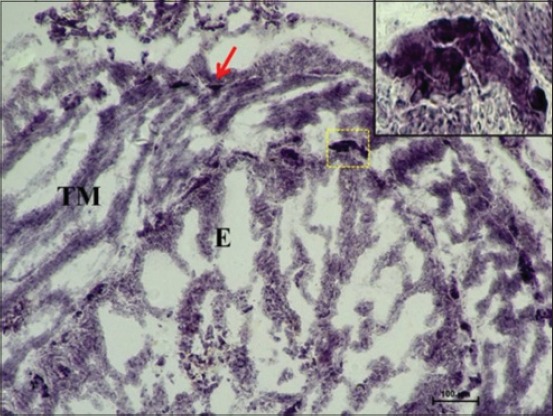
Cryostat section of ileocecal orifice 54 cm curved crown-rump length (195 days) fetus showing strong nicotinamide adenine dinucleotide-diaphorase activity in epithelium (E), tunica muscularis (TM), intense activity in submucosal lymphoid aggregates (yellow boxed; inset) and in nerve plexuses within layers of tunica muscularis (red arrow). Nitro BT method ×100.

At 54 cm CVRL (195 days), in cecum weak to moderate G-6-PD activity was observed in primordia of lymphoid tissue in submucosa and in tunica muscularis. However, the activity was reduced in the tunica mucosa specifically the columnar epithelial lining. In rats similarly, G-6-PD activity was reported to be highest in the upper small intestine, and relatively low activities were observed in the other parts of the gastrointestinal tracts of the various kinds of rats and the activity of the enzyme in germfree rats was about threefold higher than that in the conventional rats [8]. A strong activity of NADH-d and NADPH-d was noticed in the basement membrane of epithelium of villi and tunica muscularis of cecum and ileocecal orifice as well. Intense activity of this enzyme also was noticed in the primordia of the lymphoid tissue developed on the antimesenteric side observed at this age (Figure-6). Intense activity of enzyme may reflect differentiation and maturation of lymphocytes [15]. The nerve plexuses in tunica muscularis also had intense activity of this enzyme (Figure-6).

**Figure 7:**
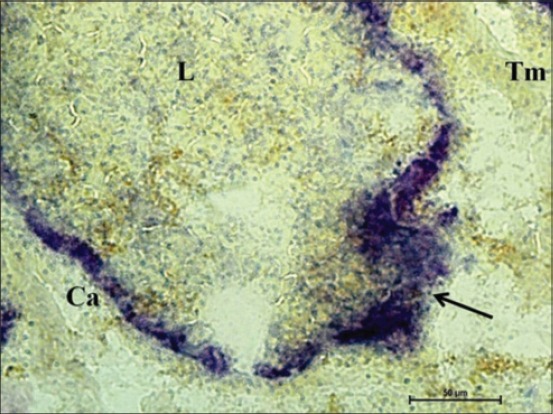
Photomicrograph of cecum of 100 cm (full term) fetus depicting strong alkaline phosphatase activity in the capsule (Ca) of the lymphoid tissue (L) at the apical region (arrow) and very weak activity in the center of lymphoid tissue and glandular epithelium of tunica mucosa (Tm). Azodye method ×400.

**Figure 8:**
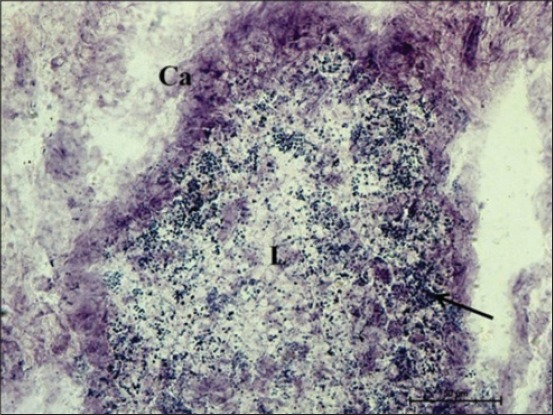
Cryostat section of cecum of 100 cm (full term) fetus presenting strong nicotinamide adenine dinucleotide-diaphorase activity in the capsular layer surrounding the lymphoid tissue, peripheral region had moderate granular activity and weak in the center of the solitary lymphoid tissue. Nitro BT method ×400.

**Figure 9:**
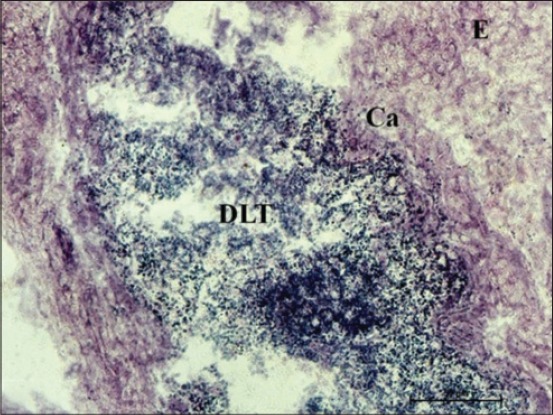
Photomicrograph of cecum of 100 cm (full term) fetus showing weak G-6-phosphate dehydrogenase activity in columnar epithelial cells (E), moderate in the capsule (Ca) and strong granular in center and cellular contents at the periphery of diffuse lymphoid issue. Nitro BT method ×400.

At 100 cm (full term), the AKPase activity was noticed as a strong reaction in the capsular covering of the completely developed lymphoid tissue, especially at the apical region, i.e. entering the tunica mucosa. The glandular epithelium of tunica mucosa at this age had a very weak activity of this enzyme (Figure-7). The greater enzyme activity of AKPase in the cranial region of the intestine of Dourado fish and lesser in the posterior region suggests less absorption of nutrients such as glucose, calcium, lipids, and inorganic phosphate in the large intestine [16]. Moreover, the center of the lymphoid tissue complex formed with the invasion of cecal glands at this age. Similar localization of DLT was observed in basal part of cecal glands, thus forming lymphoglandular complexes was observed in goat fetuses [9]. At this age activity of NADH-d and NADPH-d was strong in the capsular layer surrounding the lymphoid tissue. Moreover, center of the solitary lymphoid tissue had weak activity but its peripheral region had moderate granular NADH-d activity (Figure-8). The similar distribution of solitary nodular units was reported in cecum of chickens [6]. NADH-d is a coenzyme dehydrogenase and acts in cell as a part of hydrogen transport chain. At this age, weak activity of G-6-PD was found in columnar epithelial cells of cecum, but the capsule of lymphoid tissue had moderate activity of this enzyme. However, the capsular region specifically covering the dome of the lymphoid region had stronger activity of the enzyme. Similar findings were reported on lymphoid tissue of rabbit cecum [2]. Moreover, the cellular contents at the periphery and in the center of DLT had strong fine granular activity of this enzyme (Figure-9). Similarly, the presence of DLT was reported in chickens during their late embryonic life [7]. The G-6-PD enzyme is associated with the pentose phosphate shunt [15] and these pentose phosphates might be utilized for nucleic acid synthesis in lymphopoiesis. An overall moderate activity of NSE was observed in submucosal DLT that was fine granular toward its periphery. A mild activity of NSE was reported in the 26^th^ or 28^th^ day of fetal life in rabbit, although the activity was strong in 1 day, 19 days and older rabbits [5]. Therefore, the distribution of these enzymes throughout the cecum varies within its structural components at different gestational periods to depict their active physiological roles that can be helpful in future for differentiating health and disease conditions affecting gut.

## Conclusion

This study revealed that the distribution of enzymes was uniformly weak in villi-like projections in cecum of Group I and completely absent from submucosa. In Group II, the enzymes showed a moderate to strong activity in epithelium lining tunica mucosa which progressively decreased as the fetus progresses toward late gestational age. However, the intense activity of enzymes was observed in developing lymphoid tissue in this group. In Group III, distribution of enzymes reduced in tunica mucosa of cecum with advancing age whereas intense activity was noticed in the developed lymphoid tissue complex.

## Authors’ Contributions

KK has planned and designed the study. The collection of samples and laboratory work was done by KK. OS analyzed the data and provided technical support. The manuscript was prepared under the guidance of OS. All authors participated in draft of the manuscript, read and approved it.
